# Evaluating the effect of an educational program on increasing cervical cancer screening behavior among rural women in Guilan, Iran

**DOI:** 10.1186/s12905-020-01020-7

**Published:** 2020-07-20

**Authors:** Sedighe Bab Eghbal, Mahmood Karimy, Parisa Kasmaei, Zahra Atrkar Roshan, Roghieh Valipour, Seyedeh Maryam Attari

**Affiliations:** 1 Reproductive Health Research Center, Department of Obstetrics and Gynecology, Al-Zahra Hospital, School of Medicine, Guilan University of Medical Sciences, Rasht, Iran; 2 Department of public health, faculty of health, Social Determinants of Health Research Center, Saveh University of medical sciences, Saveh, Iran; 3 Department of Health Education and Promotion, Research Center of Health and Environment, School of Health, Guilan University of Medical Sciences, Rasht, Iran; 4 Department of Social Medicine, School of Medicine, Guilan University of Medical Sciences, Rasht, Iran; 5 Department of public health, Mazandaran Training & Education Organization, Babol, Iran

**Keywords:** Cervical cancer, Education, Pap smear test, Screening

## Abstract

**Background:**

Cervical cancer is one of the major health problems and the third prevalent cancer in women all around the world. As a simple, inexpensive, and with no side-effects, Pap test is a reliable way to screen cervical cancer. This study aimed to investigate, the effects of educational intervention based on the Health Belief Model (HBM) on doing Pap smear tests among the rural women of the north of Iran.

**Methods:**

In a quasi-experimental study, 160 rural women were randomly divided into control and experimental groups to experience a three-session intervention. The experimental group received the usual educational programs of rural health center and educational programs based on the HBM constructs through personal consultation, asking/answering questions, and an educational pamphlet. The control group, received the usual educational programs of rural health center. The post-test data were collected 2 months after the intervention and analyzed in SPSS-18.

**Results:**

Before the intervention, there was no significant difference between the control and experimental groups regarding the mean score of knowledge, performance and constructs of the HBM. After the intervention, however, there was a significant difference in the mean scores of knowledge performance and all constructs of the HBM in two groups (*p* < 0.001). Rate of doing the Pap smear test in the experimental group increased from 18.7 to 78.7% in the intervention group.

**Conclusion:**

These findings support the effectiveness of cervical cancer prevention programs based on the HBM. Therefore, conducting similar programs in other regions is recommended.

## Background

After breast and colorectal cancers, cervical cancer is the third prevalent cancer in women [1]. Each year, this disease afflicts about 300 to 400 thousand new patients and there are nearly 200 thousand deaths worldwide. Nowadays the highest prevalence of the disease is in the developing countries so that about 80% of its global cases are diagnosed in these countries [2]. About 15% of women’s malignancies in developing countries are caused by cervical cancer, this rate is about 1% in developed countries [2]. The incidence of cervical cancer in Iran is reported 3.73 per 100 thousand women. Meanwhile in Guilan province, rate of the incidence of this cancer is 0.48 per 100 thousand women [3]. According to experts, these geographical differences are mostly due to the availability or unavailability of an effective screening and a therapeutic program [2] and also these women’s inaccessibility to do the Pap smear test for early detection of this cancer [4]. The death and incidence rate of cervical cancer in most developed countries has declined due to routine Pap smear tests and recently because of Human Papillomavirus (HPV) screening [5].

The most important risk factors of this disease affliction are pregnancy in young ages, several sex partners, Human Immunodeficiency Virus [6] infection, Herpes Simplex Virus (HSV), cytomegalovirus (CMV), HPV, exposure to DES (diethylstilbestrol) during embryonic period, sexually transmitted infections, frequent infections, the immune system weakness, contraceptive medicines, diet (shortage of serum folate, vitamins C and A, and beta carotene), genetic factors, and exposure to chemical substances (for the women working in chemical plants or farms) [7–9].

Cervical cancer is recognized as a preventable cancer taking into account the long precancerous condition, accessibility to reliable screening plans, and efficient treatment of the early lesions [7, 8]. Improving the survival of cervical cancer patients significantly depends on the stage of the disease at the time of diagnosis. 5-year survival in the early stages of the disease is 92% and in advanced stages is 13% [4, 7]. The death and incidence rate of cervical cancer in most developed countries has declined due to routine Pap smear tests [5].

In total, preventive health behaviors can lead to satisfactory health results. The preventive behavior for this disease is the Pap smear test, which is a fast way to diagnose the cancer and attenuates its effects to a great extent [10]. Cervical cytology (Pap smear) is for distinguishing cervix abnormal cells from its normal cells. Cervical cancer may remain in non-invasive stage for 20 years and shed abnormal cells are detectable by cytological evaluation (Pap smear). In case of early detection and timely treatment, 40–60% of dysplasia (the abnormal growth or development of a tissue) cases regress and the rest progress to invasive cancer. With the increasing use of Pap smear in different countries cervical cancer deaths are reduced about two third [11].

Despite the remarkable success of the Pap smear test in cervical cancer diagnosis, participation rate in developing countries is only 5%, while in high-income countries such as the USA this rate is about 90%. In Iran, several studies have reported low participation rate in the test. For instance, this rate in study of Babazade et al. [12] was reported 27.1% and in study of Farzane et al. [13] was reported 50%. In the case study province (Guilan) this rate was 0.31% and in Shaft county was 17.59%.

As a healthy behavior and a way to improve health conditions, the Pap smear test is a screening test for cervical cancer in women who demonstrate no symptoms. This test is conducted every 3 years on women who were or are sexually active [14].

Pap smear test is considered as the most efficient and economical way for screening the cancer and a simple, economic and with no side-effect way for screening cervical cancer [15]. If performed properly and with proper sampling tools, Pap smear test can detect cervical cancer with an accuracy of 70–95% [9]. Lack of regular Pap smear test screening leads to a 2–6 times increase in the risk of cervical cancer [8]. About 70% of women who die from cervical cancer have not done Pap smear test regularly [9]. Using appropriate models and theories is the first step in the process of programming for health education. Based on the different studies results, the HBM is an appropriate educational model. It is a comprehensive model that rather than controlling the disease, is mostly used for its prevention and emphasizes on how one’s perceptions cause motivation and movement, and lead to behavior [16]. According to the HBM, to adopt cervical cancer preventive functions, the individual needs to believe that they are prone to a disease like cervical cancer (perceived sensitivity, such as I am worried about my suffering from cervical cancer), perceive the seriousness of the different side effects of the disease on the different aspects of their lives (perceived severity, such as if cervical cancer is not diagnosed and treated in time, causes my death), and find recommended behaviors like Pap smear test, effective in attenuating the risk or severity of the disease (perceived benefits, such as Pap smear test makes me be sure about my healthiness) to overcome the action barriers like time, cost, pain, etc. (perceived barriers such as I do not do Pap smear test because I am afraid of its result).

Also, the individual needs to believe in their ability to do a successful Pap smear test (perceived self-efficacy, such as I am sure I can do Pap smear test regularly according to sanitary staff’s recommendations) to be able to perform a risk preventive function – i.e. Pap smear test [17, 18] .Taking into account that awareness of early symptoms, early detection, and timely treatment are critical to cancer control and that studies have shown that Pap smear test in Iran is notably less common than other countries, therefore there is a serious need for effective educational intervention. The educational intervention results based on HBM show that education according to the model constructs has significantly increased women’s referrals to do the Pap smear test [19, 20]. Therefore, and given the above introduction, the present study is an attempt to determine the effects of the HBM-based education on conducting Pap smear tests on the women living in rural areas of Shaft, Guilan, Iran.

## Methods

### Design

Women living in rural areas covered by Shaft health center (Guilan, Iran) participated in a quasi-experimental study from November 2017 till April 2018.

### Sample and setting

The participants were selected by a two-stage random sampling method. To this end, a rural health center among the rural health centers affiliated with Shaft health center was selected randomly. Then six sub-centers (called health houses in Iran) affiliated with the selected rural health center constituted the study group. All the health houses one by one and by the simple random method were divided into experimental and control groups. Three of the six health houses were selected as the intervention group and other three were selected as the control group. In the second step, going to health houses and using family rosters available there, eligible subjects were selected by systematic sampling method $\left(\frac{960}{160}\right)$ (in other words, every 6th subject was selected) so that 27 women from the first and fifth health houses and 26 women from the third health house were selected as the subjects in the intervention group and 27 women from the second and fourth health houses and 26 women from the sixth health house were selected as the subjects in the control group.

By using Pocock’s formula and considering mean changes, standard deviation of the self-efficacy construct in previous similar study, reliability coefficient of 95%, accuracy of 5%, Z_1-α/2_ = 1.96, Z_1-β_ = 1.28, = α 0.05 and β =0.1 sample size was calculated *n* = 66.4.

$$ n=\frac{{\left[{\mathrm{Z}}_{1-\alpha /2}+{\mathrm{Z}}_{1-\upbeta}\right]}^2\left({S}_1^2+{S}_2^2\right)}{{\mathrm{d}}^2} $$

**Fig. 1:**
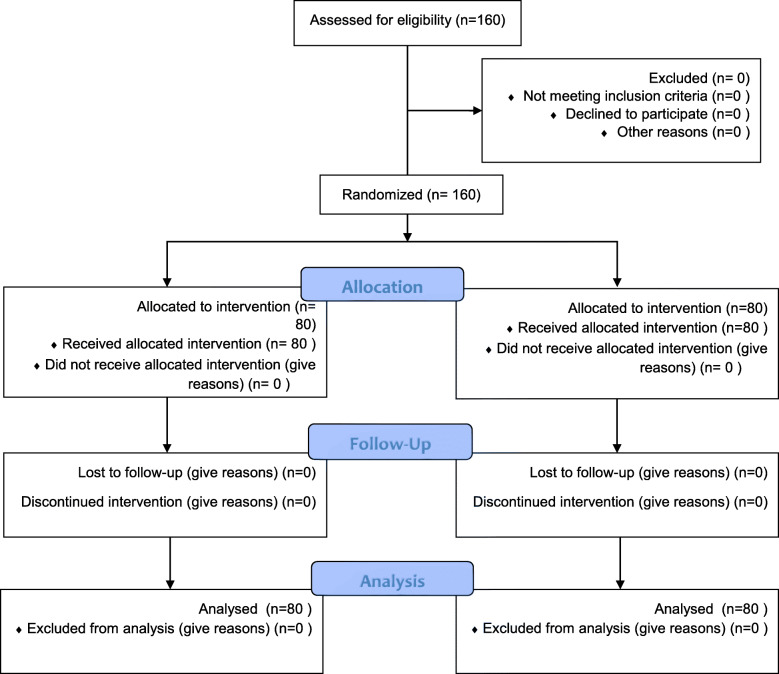
Consort diagram of the participants

To increase the study power and given the probable leaves, the sample size was increased by 20% so that 160 individuals were selected totally (80 subjects for each group). All the subjects in intervention and control groups were assessed in terms of inclusion and participation criteria. Also all the subjects allocated to educational intervention program participated in the intervention, and none of them in both intervention and control groups discontinued the program so that all of the subjects in both groups were included in the analysis. (Fig. 1).

Inclusion criteria were 20–65-year-old women married at least once [21]. Married women who have been married for at least 6 months and have no history of hysterectomy and no history of cervical cancer. Exclusion criteria were a reluctance to participate and missing two educational sessions.

### Measures

Data gathering tool was a multi-item questionnaire with three sections including demographics, knowledge, and the HBM constructs used in similar researches [6, 22]. After holding research team meetings, and validity and reliability measurement, final questionnaire combination of was compiled and used.

The first section (demographics) consisted of 12 questions and the second section (knowledge) consisted of 23 knowledge questions (score range 0–42) with 1 point for correct answers, and 0 point for incorrect answers; Section three (the HBM constructs) consisted of 35 items including perceived sensitivity (six items; score range 6–30); perceived severity (five items; score range 5–25); perceived benefits (five items; score range 5–25); perceived barriers (14 items; score range 14–70); perceived self-efficacy (six items; 5–25). The answers were designed on Likert five-point scale from 1(totally disagree) to 5 (totally agree).

Pap smear test function was assessed by one question. Besides, using a checklist, women’s medical file was checked in terms of doing Pap smear test before and after the educational intervention. The validity of the tool was examined by using a content validity test, content validity index (CVI) and content validity ratio (CVR). To this end, the tool was presented to nine experts and faculty members (four health education experts, one health services management expert; one epidemiology expert, three gynecologists and midwives) at Guilan and Saveh University of Medical Sciences. They were asked to give us feedback given the objectives of the study and the relevance of the questions. From the expert’s feedback further modifications were performed on the tool so that CVR and CVI were obtained 1 and 0.90 respectively.

To check the reliability of the tool, it was provided to 20 women of the study population who were identical to the sample group in terms of demographical variables. As to the questions about knowledge, the split-half method was used and as to the HBM constructs Cronbach’s alpha was used. In the split-half method, Spearman-Brown’s coefficient was 0.71 and Cronbach’s alpha for perceived sensitivity, perceived severity, perceived benefits, perceived barriers, and perceived self-efficacy were obtained 0.71, 0.71, 0.72, 0.85 and 0.86 respectively.

Then the participants signed a written consent. Also the participants filled in the questionnaire in two stages before and after the educational intervention in a separate room at the health house. For illiterate and almost illiterate participants, the questionnaire was completed by trained people and through interview. The educational sessions were held at the same place too.

#### Intervention

Based on the pretest data analysis, and regarding the predictive power of knowledge, perceived severity and perceived benefits constructs with behavior in logistic regression, 50–60-min educational sessions were designed for experimental group as follows, and were held at the health house. Educational programs were held based on the HBM (with emphasis on and allocation of more time to knowledge constructs, perceived severity and perceived benefits) during 3 weeks (each week one session). To maintain the permanence of education, a pamphlet on the benefits of doing Pap smear test was designed and given to the participants in the experimental group. Furthermore, 5 weeks after the intervention, a phone call was made and some health messages (each week one SMS) were sent to the participants in the experimental group about the risks of not doing the Pap smear test, benefits of doing it, how and where to do it (As a reminder, a warning of cues to action and tracking women in experimental group who did not do the Pap smear test) (Table 1). On the other hand, the control group received only the usual interventions and educational programs of the rural health center.

**Table 1:** Details of educational content based on the HBM

Educational sessions	HBM constructs	Teaching method	Objectives	Session content	Materials and teaching aids
Session 1	Knowledge	Lecture, Asking/answering questions	To increase the knowledge and create health awareness	Introduction, Signing a written consent consciously, Information about reproduction system anatomy, Age range to do the test, Symptoms and preventions	Slides, Pamphlet, Poster
Session 2	Perceived sensitivity	Lecture, Asking/ answering questions	Misconceptions correction, Expressing seriousness, Risk of affliction as well as the negative consequences of cervical cancer in order to increase perceived severity	Consequences of failure to observe reproduction organs hygiene, Physical and emotional consequence of cervical cancer, The effects of cervical cancer on one’s job, family and chance of having children	Film, Pamphlet, Poster
	Perceived severity	Lecture, Film, Asking/ answering questions			
Session 3	Perceived barriers	Group discussion, Brainstorming	Familiarity with the benefits of the proposed methods to attenuate risk or the severity of cervical cancer	Benefits of doing Pap smear test and its effects on physical and psychological health, Benefits of early detection through screening, Risk attenuation methods, Stress attenuation methods, Strategies, Alternative behaviors to promote Pap test	Pamphlet, Poster, Memory cards, Scheduling cards
	Perceived benefits	Lecture, Group discussion, Brainstorming, Using motivations			
	Perceived self-efficacy	Motivating, Boosting, Reducing stress			

### Statistical analysis

Two months after the educational intervention, the participants in the two groups filled out the questionnaire once more, and the collected data were analyzed in SPSS 18. The data analysis was done using descriptive statistics such as definite and relative frequency distribution, mean indices and standard deviation, and inferential statistics such as independent t-test, Mann Whitney U, Wilcoxon, and paired t-test. Besides, the Chi-square test was used to measure variation in performance before and after the intervention.

#### Ethics

prior to any study activities, a written informed consent form was obtained from all the participants of the study. All participants were given an information sheet together with the informed consent with the advice that they could revoke their consent at any time without giving any reasons. After securing an approval letter from the Research and Technology Department, the study was approved by the Ethics Committee under No. 4920341301 and registered on the Iran Clinical Trial database (IRCTID: IRCT2013123016006N1). The participants in both groups were ensured that their information would remain confidential throughout the study.

## Results

One hundred sixty rural women between the ages 20 and 65 took part in this study. The mean age of the participants in the experimental and control groups was 42 ± 10.8 and 40 ± 11.4 respectively. The marriage age of the majority of women in the experimental (85%) and control (88.8%) groups was 17 and more. The number of child deliveries of the majority of women in experimental (68.8%) and control (78.8%) groups was between zero and three. The majority of the participants in the experimental group were illiterate (36.2%) or had elementary education (36.2%); these figures in the control group were 26.2 and 36.2% respectively. The majority of the participants’ husbands in the experimental group (46.2%) and control group (37.5%) were farmers and all the participants were homemakers. According to the Chi-square test, the experimental and control groups were identical in terms of demographic variables and there was no significant difference between them (*p* > 0.05) (Table 2).

**Table 2:** Comparison of qualitative variables in two groups of Intervention and Control women

Variables	Intervention		Control		*P*-value *
	Number	Percentage (%)	Number	Percentage (%)	
**Age**					
≤ 30	10	12.5	17	21.2	0.264
31–40	28	35	31	38.8	
41–50	17	21.2	16	20	
> 50	25	31.2	16	20	
**Marital status**					
Married	75	96.2	73	93	0.468
single	5	3.8	7	6.2	
**Marriage age**					
< 17	12	15	9	11.2	0.428
≥ 17	68	85	71	88.8	
**Education**					
Illiterate	29	36.2	21	26.2	0.455
Elementary	29	36.2	29	36.2	
Middle school	12	15.1	15	18.8	
High school and diploma	10	12.5	15	18.8	
**Number of deliveries**					
0–3	55	68.8	63	78.8	0.151
≥4	25	31.2	17	21.2	

*Chi-square

More than 90% of the women in the study knew about one of the risk factors of cervical cancer (the participants were asked to designate the most important risk factor of cervical cancer from their own point of view) (Table 3). There was no significant difference between the two groups before the intervention in terms of knowledge and perceived sensitivity, severity, benefits, barriers, and self-efficacy (*P* > 0.05). However, after the educational intervention there was a significant difference between the two groups in terms of the mean scores of all the above-mentioned constructs (*P* < 0.001). In other words, after the educational intervention, the mean scores of knowledge and all the constructs of the HBM increased significantly and the mean score of perceived barriers decreased significantly in the experimental group; while there was no significant difference in the mean scores of constructs in the control group (*p* > 0.05) (Table 4).

**Table 3:** Knowledge frequency distribution of women under study according to each risk factor of cervical cancer

Risk Factors of Cervical Cancer	Yes N (%)	No Idea and No N (%)
Marriage at an early age (under 16)	101 (63.1)	59 (36.8)
First pregnancy at an early age (under 20)	104 (65)	56 (35)
High number of deliveries (4 and more)	109 (68.1)	51 (31.8)
Women whose husbands had a wife with cervical cancer	107 (66.9)	53 (33.1)
Women whose husbands have multiple spouses	108 (67.5)	52 (32.5)
Deficiency of Vitamin A, C and Folic Acid	114 (71.2)	46 (28.7)
Women who have been married more than once	121 (75.6)	39 (24.3)
Women who smoke	137 (85.6)	23 (14.3)
Family history of cervical cancer	126 (78.8)	34 (21.2)
Taking contraceptive pills	72 (45)	88 (55)
Non-compliance with genital hygiene	149 (93.1)	11 (6.8)
One of the couples’ history of STDs	138 (86.2)	22 (13.7)
Low socioeconomic status	150 (93.8)	10 (6.2)

**Table 4:** Comparison of HBM constructs in two groups at before and after of intervention

Variable	Group Time	Intervention group Mean ± SD	Control group Mean ± SD	*P*-value*
**Knowledge**	Baseline	20.5 ± 2.2	20.1 ± 1.8	0.580
	2-months follow-up	25.2 ± 2.1	19.7 ± 1.6	0.001
	*P*-value**	0.001	0.435	–
**perceived sensitivity**	Baseline	22.5 ± 2.7	22.6 ± 2.1	0.101
	2-months follow-up	29.0 ± 2.20	22.7 ± 2.9	0.001
	*P*-value**	0.001	0.490	–
**perceived severity**	Baseline	19.6 ± 1.7	19.8 ± 1.0	0.802
	2-months follow-up	24.5 ± 1.1	19.6 ± 1.7	0.001
	*P*-value**	0.001	0.329	–
**perceived benefits**	Baseline	20.1 ± 1.9	19.3 ± 1.3	0.181
	2-months follow-up	24.2 ± 1.6	19.2 ± 1.4	0.001
	*P*-value**	0.001	0.469	–
**perceived barriers**	Baseline	30.9 ± 5.6	29.8 ± 3.5	0.359
	2-months follow-up	18.0 ± 6.5	30.1 ± 3.7	0.001
	*P*-value**	0.001	0.07	–
**perceived self-efficacy**	Baseline	20.1 ± 3.3	19.8 ± 1.1	0.06
	2-months follow-up	24.7 ± 1.0	19.1 ± 3.0	0.001
	*P*-value**	0.001	0.06	–

* Independent T-test; ** Paired T-test

As listed in Tables 5 & 6, the rates of doing Pap smear test before the educational intervention in the experimental and control groups were 18.7 and 16.2% respectively. These figures after the educational intervention increased significantly in the experimental (78.7%) and control (22.5%) groups (*p* < 0.001).

**Table 5:** Frequency distribution of Pap smear test before the educational intervention

Variables	Intervention		Control		*P*-value*
	Number	Percentage (%)	Number	Percentage (%)	
Pap smear test					
Yes	15	18.75	13	16.25	0.836
No	65	81.25	67	83.75	

*.Fisher’s Exact Test

**Table 6:** Frequency distribution of Pap smear test after the educational intervention

Variables	Intervention		Control		*P*-value*
	Number	Percentage (%)	Number	Percentage (%)	
Pap smear test					
Yes	63	78.75	18	22.5	0.001
No	17	21.25	62	77.5	

*. Fisher’s Exact Test

## Discussion

The health education program based on the HBM improved the performance in the intervention group in terms of doing Pap smear test screening. Although, the routine educational programs of the health center increased the rate of Pap smear test in the control group, this increase in the experimental group was much higher and close to 80%. The significant increase in the rate of doing Pap smear test after the educational intervention based on the HBM has been also reported by Shobeiri et al. [10], Parsa et al. [23], Koc et al. [24], and Kolutek et al. [25],Taking into account the importance of pap smear test and the role of health centers in this regard, it seems that implementation of such programs based on the HBM in clinics and health centers may lead to an increase in the quality of education, more effectiveness in the target group, creation of higher motivation in the individuals to attend the screening program, and higher chance of early detection of pre-cancer and cancer lesions. All these result in a less prevalence of cervical cancer, fewer medication costs, and lower death rates.

The educational intervention improved the women’s knowledge score in the experimental group about the cervical cancer. Consistently, Bebis et al. [26], and Shobeiri et al. [10], showed that after the educational intervention, the mean score of knowledge has been improved. Other studies have shown that there is a significant and direct relationship between awareness level and performance so that with higher awareness, the chance of doing Pap smear test increases. For instance, Lee et al. [27] studied Korean women and showed that one of the barriers to do the Pap smear test was the low level of knowledge in women. Karimy et al. [28], showed that lack of knowledge or positive attitude were some of the barriers to healthy behaviors for doing Pap smear tests. The educational intervention was effective in creating mental belief in the experimental group subjects about damages caused by cervical cancer, risk perception of affliction of this disease, seriousness of the disease, costs and treatment hardships for higher perceived sensitivity and severity. The increase of the mean scores of perceived sensitivity and severity after the intervention is compatible with other studies that have used the same model [10, 23]. McFarland et al. [29], and Demirtas et al. [30] mentioned that the reason for not doing the Pap smear test was the low perceived sensitivity and severity in women. According to the HBM, to create motivation for doing a healthy behavior, the individual needs to know that they are at risk of affliction of a health problem. To have an effective education, trainers need to describe the probability of creation of a health problem, intensifying the risks, creation of sensitivity and increase in perceived severity of the problems, therefore good grounds for action are created.

The participants’ perception of benefits and barriers of doing Pap smear test in the two groups before the educational intervention, was identical. However, after the intervention, the perceived benefits increased and perceived barriers decreased in the experimental group.

similar to our findings, Park et al. [31], reported that after education, the experimental group had a higher score of perceived benefits of Pap smear test. Garces-Palacio et al. [32], and Jirojwong et al. [33], argued that there was a positive relation between the perceived benefits and the rate of doing Pap smear tests. According to the HBM, an individual performs an action when they rationally find its benefits more than the barriers. In other words, people do not necessarily accept health recommendations unless they clearly understand the possible benefits of such behavior than its barriers. Other studies have shown that perceived barriers are among the main constructs related to Pap smear test. De Peralta et al. [34], and Chesun et al. [35], argued that the perceived barriers were the main predictors of the screening behavior. Similarly, Demirtas et al. [30], argued that the probability of doing a Pap smear test was higher when women had lower perceived barriers. This means that lowering the barriers leads to a higher rate of doing Pap smear tests.

Our results showed, the mean score of perceived self-efficacy in the experimental group increased after the intervention compared with the control group. This result is similar to other studies. High self-efficacy increases one’s ability, capability, competence and self-confidence to demonstrate behavior successfully [36]. Researchers believe that people with higher self-efficacy tend to show higher persistence and hardworking attitudes confronting hardships. Improvement in individuals’ self-efficacy enables them to overcome challenges and hardships easier, which is reflected in their performance [23, 36, 37]. Taking into account the role of this construct in enabling women to adopt healthier behaviors, it should receive more attention in designing educational programs.

Although these findings support the effectiveness of the educational intervention, this study had some limitations including the effects of other information sources on the experimental and control groups. However, this research had some strengths too, that using the HBM has helped the evaluation of attitude and behavior and also making educational intervention was effective in creating healthy behavior in rural and deprived women.

## Conclusions

Health educational program based on the HBM, improved knowledge and performance of the women under study as for doing Pap smear test. Therefore, designing and implementing educational interventions using the HBM in health centers is recommended. Other recommendations are a routine assessment of continuity of behavior change with longer follow-ups (more than a year) and conducting similar studies with other behavior change models. Conducting similar studies using the HBM, especially perceived severity and benefits constructs and also other behavior models to find the best model to persuade women in different cultures to do the Pap smear test in developing countries is recommended.

## Data Availability

Data for this study were extracted from a MSc dissertation at the Guilan university of Medical Sciences. All data generated during and/or analyzed during the study are available by the correspondent author on reasonable request.

## Abbreviations

HBM: Health Belief Model
HPV: Human papillomavirus
CVI: Content Validity Index
CVR: Content Validity Ratio
HIV: Human Immunodeficiency Virus
HSV: Herpes Simplex Virus
DES: Diethylstilbestrol
CMV: Cytomegalovirus
